# Fluorescence Polarization Immunoassay of Mycotoxins: A Review

**DOI:** 10.3390/toxins1020196

**Published:** 2009-12-10

**Authors:** Chris Maragos

**Affiliations:** Bacterial Foodborne Pathogens and Mycology Research Unit, US Department of Agriculture, Agricultural Research Service, 1815 N. University St., Peoria, IL 61604, USA; Email: chris.maragos@ars.usda.gov; Tel.: +1-309-681-6266; Fax: +1-309-681-6689

**Keywords:** fluorescence polarization immunoassay, mycotoxins, review

## Abstract

Immunoassays are routinely used in the screening of commodities and foods for fungal toxins (mycotoxins). Demands to increase speed and lower costs have lead to continued improvements in such assays. Because many reported mycotoxins are low molecular weight (below 1 kDa), immunoassays for their detection have generally been constructed in competitive heterogeneous formats. An exception is fluorescence polarization immunoassay (FPIA), a homogeneous format that does not require the separation of bound and free labels (tracer). The potential for rapid, solution phase, immunoassays has been realized in the development of FPIA for many of the major groups of mycotoxins, including aflatoxins, fumonisins, group B trichothecenes (primarily deoxynivalenol), ochratoxin A, and zearalenone. This review describes the basic principles of FPIA and summarizes recent research in this area with regard to mycotoxins.

## 1. Introduction

Mycotoxins are low molecular weight (less than 1 kDa) toxins produced by fungi in a wide variety of commodities and foods. Several of the mycotoxins are capable of causing diseases in animals and represent a potential hazard for humans as well [1]. Because mycotoxins are relatively small molecules they have generally been detected using competitive, rather than non-competitive, immunoassays. Furthermore, most of the competitive assays are surface-based. That is, they require either a toxin-protein conjugate or an antibody to be immobilized onto a surface (membrane, well, electrode, sensor surface, *etc*.). This is done to facilitate separation of the bound and unbound forms of the competing reagents. In typical competitive enzyme-linked immunosorbent assay (ELISA) formats the signal developed depends upon the presence of an enzymatic tracer. Generally the tracer is either the toxin that has been labeled with an enzyme (often used in cases where antibody is immobilized) or antibody labeled with an enzyme (in cases where a toxin-protein conjugate is immobilized). The same two configurations have been used in many immunoassays and biosensors. Non-enzymatic labels such as fluorescence, radioisotopes, colloidal gold, *etc*. have also been used to facilitate detection of the competitive event. Assays of this nature, which require separation of the ‘free’ and ‘bound’ tracer are termed heterogeneous and encompass the vast majority of mycotoxin immunoassays. The separation can be achieved in various ways, from chromatographically (as in lateral flow test strips), washing (as in ELISAs), or reagent flow over a surface (as in certain biosensors). The various types of mycotoxin immunoassays were recently reviewed [2], and many mycotoxin immunoassays can be purchased commercially.

**Figure 1:**
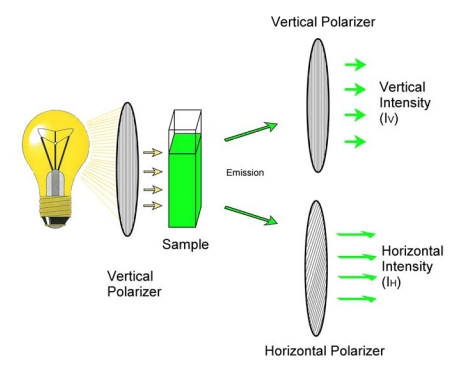
Measurement of fluorescence polarization.

Fluorescence polarization immunoassay (FPIA) differs from ELISA in that it is a homogeneous assay conducted in solution phase. Unlike heterogeneous immunoassays, homogeneous assays do not require the separation of the free and bound tracer. This has the potential to be a significant advantage, particularly if it eliminates the need for additional manipulations, such as the washing steps of competitive ELISAs. When a fluorophore in solution is exposed to plane-polarized light at its excitation wavelength the resulting emission is depolarized. The depolarization results from the motion of the fluorophore during the processes of excitation and emission. Because of this, the more rapid the motion of the fluorophore the more the emission is depolarized. The fluorescence emission can be segregated, using polarizers, into horizontal and vertical components, shown schematically in Figure 1.

In its simplest sense the polarization can be expressed as the ratio of the difference in emission in the vertical (I_V_) and horizontal (I_H_) planes divided by their sum. That is, where P is the polarization of the emission, P= (I_V_ - I_H_)/(I_V_ + I_H_) [3,4]. The polarization is often expressed in polarization units or millipolarization units (mP). An interesting aspect of fluorescence polarization is that P is not dependent upon the absolute intensity of the fluorescence, but rather the relative intensity of the components of that fluorescence. As such, different concentrations of the same fluorophore (with different absolute intensities) can give rise to the same polarization value. Environmental factors that influence the molecular motion of the fluorophore have the potential to influence the polarization. Examples include temperature, viscosity, and the presence of materials that bind to the fluorophore. The latter property is especially important to FPIA. 

**Figure 2:**
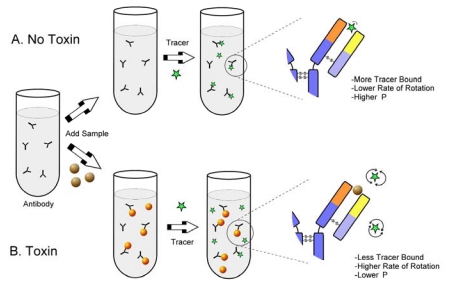
Fluorescence polarization immunoassay.

As a relatively large molecule such as an intact IgG (MW approximately 150 kDa) binds to a small fluorophore (less than 1 kDa) the rate of the tumbling motion of the fluorophore is reduced, resulting in an increase in observed polarization. The basis for competitive FPIA is shown in Figure 2. In order to make the assay specific for a toxin, the toxin can be covalently linked to the fluorophore to make a fluorescent tracer [5]. In this case the tracer competes with toxin (from the sample) for a limited amount of toxin-specific antibody. In the absence of toxin the antibody binds the tracer, restricting its motion and causing a high polarization. In the presence of toxin less of the tracer is bound to the antibody and a greater fraction exists unbound in solution, where it has a lower polarization. 

With this format the polarization is inversely related to the toxin concentration. The advantage of this format, relative to a competitive ELISA format, is the lack of a need to separate the free from the bound tracer, potentially improving assay speed. Development of competitive FPIA for mycotoxins requires a fluorescent tracer (usually a fluorescein conjugate of the toxin), a high molecular weight material to bind the toxin (generally an antibody), and an instrument to measure the polarization. Commercial fluorescence polarization instruments are readily available from several manufacturers and include benchtop models, portable models, and microplate readers. The application of FPIA to a wide variety of small molecules was summarized recently by Smith and Eremin [6]. 

Assays can be performed in several ways, one of which is shown in Figure 2. A cuvette is filled with dilute antibody solution, a portion of sample extract is added and the fluorescence intensities (I_V_, I_H_) of the blank are obtained. The tracer is then added, mixed, and held for a period before re-introducing the cuvette into the instrument to obtain the fluorescence polarization measurement. The holding period, generally ranging from several seconds to several minutes can be an important factor in the assay, as will be discussed further below. FPIA for most of the major mycotoxins have been reported. These are summarized in Table 1.

**Table 1:** Literature on FPIA of mycotoxins.

Toxin	Analogs tested	Tracer^a^	Matrix	Sample Cleanup	Limit of Detection or IC_50_	Ref.
Fumonisins	FB_1_, FB_2_, FB_3_	FB_1_-DTAF	maize	Filtration, Dilution	LOD: 500 μg/kg in maize	[7]
Aflatoxins	AFB_1_, AFB_2_, AFG_1_, AFG_2_	AFB_1_-FL	Maize, sorghum, peanut butter, peanut paste, popcorn	Filtration	IC_50_: 28 ng/mL (AFB_1_) in methanol/water	[8]
Ochratoxin A	OTA	OTA-EDF	barley	Centrifuge, Filtration, Dilution	LOD: 3 ng/mL in buffer	[9]
	OTA	OTA-EDF	rice	Centrifuge, Dilution	LOD: 0.3 ng/mL in buffer	[10]
	OTA	Oligo-Fluo	buffer	none	LOD: 2 ng/mL in buffer	[12]
	OTA	OTA-EDF	red wine	Dilution, SPE^b^	LOD: 0.7 ng/mL in red wine	[11]
Deoxynivalenol	15-Ac-DON, DON, HT-2 toxin	DON-FL	wheat	Centrifuge	IC_50_: 30 to 1000 ng/mL in buffer (see text)	[13]
	3-Ac-DON, DON, 15-Ac-DON	DON-FL2	wheat, maize	Filtration	IC_50_: 12 ng/mL in buffer	[14]
	3-Ac-DON, DON, 15-Ac-DON	DON-FL2	Wheat, semolina, pasta	Filtration	LOD: 0.08 μg/kg in all three matrices	[16]
Zearalenone	ZEN, ZAN, α-ZAOL, α-ZEOL, β-ZEOL, β-ZAOL	ZEN-FL2	maize	Filtration	LOD: 110 μg/kg in maize	[17]
	ZEN, ZAN, α-ZAOL, α-ZEOL, β-ZEOL, β-ZAOL	ZEN-FL2	buffer	none	IC_50_: 67 to 450 ng/mL in buffer (multiple antibodies)	[18]
	ZEN	ZEN-HMDF	Cereal products	Filtration, dilution	LOD: 137 μg/kg in maize	[19]

^a^DTAF: 6-[{4,6-dichlorotriazin-2-yl}amino] fluorescein; FL:fluoresceinamine; EDF: fluoresceinthiocarbamyl ethylenediame; Oligo-Fluo: oligo Fluo1.12.2-4; FL2: 4’-(aminomethyl) fluorescein; HMDF: fluoresceinthiocarbamyl hexamethylenediamine.

^b ^SPE: solid phase extraction.

## 2. FPIA for Mycotoxins

### 2.1. Fumonisins

Fumonisins are mycotoxins that have been found in a variety of commodities, including maize. They have been implicated as causative agents in a pulmonary edema syndrome in swine, and in a leukoencephalomalacia syndrome in horses. Fumonisins were also carcinogenic to rodents in laboratory trials. The first reported mycotoxin FPIA application was for fumonisins in maize [7]. Two methods for data collection and analysis were examined. In the first, the fluorescence intensities of samples (I_V_, I_H_) were collected before the addition of the tracer and were subsequently used to correct the values obtained after addition of the tracer. In this manner each sample provided its own background correction. Responses from samples were compared relative to a calibration curve in buffer. In the second method, the fluorescence intensities from a buffer solution (rather than each sample) were used to correct for the background fluorescence from the samples. In the latter format the data were transformed to yield a standard curve with the maximum value of 1 and a minimum value of zero. FP data from samples were likewise transformed and used, along with the calibration curve in buffer, to determine the fumonisin content. Excluding extraction steps, the assays took approximately two minutes to complete. Recoveries from maize spiked over the range from 0.5 to 20 μg FB_1_/g averaged 94.3% with a relative standard deviation of 14.6%. Both methods were compared to an HPLC method with good agreement for both formats, but slightly better agreement for the first format. Both methods tended to overestimate toxin content at lower levels of contamination. The better performance of the first format may be due to better background correction, as each sample was essentially measured twice (first to provide the background and second after the tracer was added).

### 2.2. Aflatoxins

Aflatoxins, because of their potency as carcinogens are of relevance to human and animal health at lower concentrations than many of the other mycotoxins, and the regulatory levels for these toxins in foods and feeds are also lower (ppb level). For this reason assays for these toxins likewise need to detect lower levels. A sensitive FPIA for aflatoxins in naturally contaminated maize, sorghum, peanut paste, and peanut butter was reported [8]. The tracer was an aflatoxin-fluorescein conjugate and the incubation time was 15 min. With aflatoxin standard solutions the midpoint of the inhibition curves (IC_50_’s) for aflatoxins B_1_, B_2_, G_1_, and G_2_ were 28, 90, 100, and 100 ng/mL respectively. The assay was compared to an HPLC reference method and showed a good correlation coefficient (*r*^2^ = 0.97) for naturally contaminated samples, although there was a bias towards underestimation in (spiked) crushed popcorn. 

### 2.3. Ochratoxin A (OTA)

Ochratoxins are of interest because of their nephrotoxicity, and have received considerable attention in part because of the wide variety of food sources in which they can be detected. As with the aflatoxins, there is a need to detect OTA at part per billion (ppb) levels, which can make detection by FPIA challenging. FPIA for OTA are summarized in Table 1. Three of the reports used antibodies while one used an OTA-binding aptamer. Shim *et al*. [9] screened six OTA monoclonal antibodies and selected one for development of a FPIA for testing of barley. The dynamic range was from 5 to 200 ng/mL OTA, with an IC_50_ of 30 ng/mL. Assay times, exclusive of extraction were reported to be 7 to 10 min. Recoveries from barley spiked at 50, 100, and 500 ng/g were 91, 90, and 97% respectively. The method was tested with 120 naturally contaminated samples of barley. There was some disagreement between the FPIA and ELISA, with generally higher levels obtained by the ELISA method, which the authors suggest may be due to greater matrix interferences with the ELISA. A similar tracer was used in the development of an FPIA for OTA in unpolished rice, with an IC_50_ in buffer that was ten-fold lower [10]. Recoveries from rice spiked at 10, 50, and 100 ng/g were 108, 90, and 110% respectively. However the FPIA results for naturally contaminated samples were in poor agreement with those observed with an HPLC reference method, with a tendency for FPIA results to be higher. Recently an FPIA method has also been developed to detect OTA in red wine [11]. The wine was treated with methanol and the OTA isolated using a solid phase extraction (SPE) procedure. Assays could be performed in less than 10 min, and recovery from wine spiked with 2 and 5 ng/mL OTA averaged 79%. The FPIA method compared well to an HPLC reference method for 154 samples of naturally contaminated, and spiked, red wines. Lastly, there is also a fluorescence polarization assay for OTA that does not involve the use of antibodies [12]. Technically this is not an immunoassay, although the operational principles are similar to FPIA. In this case the OTA binding material was a single stranded DNA oligonucleotide (aptamer) that was also capable of binding to a fluorescently labeled oligonucleotide (the tracer). The OTA was detected by it’s ability to displace the labeled oligonucleotide from the aptamer. The detection limit for OTA in buffer was 5 nM (approximately 2.0 ng/mL). Neither *N*-acetylphenylalanine nor warfarin affected the interaction between OTA and the aptamer. The technique was not applied to foods, but does suggest that, in the future, binding materials other than antibodies may also be feasible in fluorescence polarization assays.

### 2.4. Deoxynivalenol

**Figure 3:**
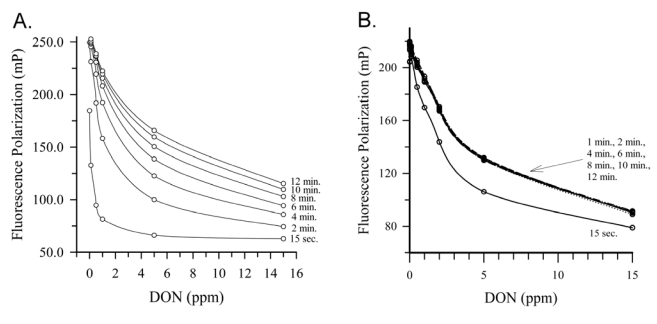
Impact of antibody and tracer choice on FPIA kinetics (a) Response with DON Mab #4 and DON-FL tracer. (b) Response of DON Mab #22 and DON-FL2. Figures reprinted by permission of the publishers [13,14].

Deoxynivalenol (DON), also know colloquially as ‘vomitoxin’, is routinely found in cereal grains worldwide and many countries have established guidance levels for this toxin in commodities and foods. Several FPIA have been reported for DON in commodities and food products (Table 1). Initial work focused on development of antibody/tracer combinations in order to reduce assay time and improve reproducibility [13,14]. These two articles described the comparison of combinations of 3 monoclonal antibodies and two tracers: the antibodies each having different cross-reactivity patterns and the tracers having been made with different fluorescein derivatives. The first of these [13] was applied to the detection of DON in naturally contaminated wheat and compared favorably to an HPLC reference method (correlation coefficient *r*^2^ = 0.97). However, it was noted that the sensitivity of the assay worsened as the tracer incubation time was lengthened. This effect is shown in Figure 3A. With a tracer incubation of 15 s, the IC_50_ for the assay was 30 ng/mL, but with an incubation of 10 min this rose to approximately 1,000 ng/mL. Although this has an advantage (the quicker assay is more sensitive) it also has a disadvantage: good control of the length of incubation was needed for good reproducibility. In the second article from the same group [14], a different antibody/tracer combination was used, which resulted in a much more rapid equilibration of the competition reaction. This is illustrated in Figure 3B, where equilibrium was obtained for the assay within 90 s. Although less sensitive than the previous assay, the rapid equilibration helped provide additional robustness to the assay. The rapid equilibration, combined with a rapid (3 min) extraction of DON from wheat or maize yielded a rapid assay (approximately 10 min, including extraction). Recoveries from spiked wheat over the range from 0.5 to 10 μg/g averaged 71%, compared to 91% by an HPLC reference method. Recoveries from spiked maize averaged 94%. The FPIA and HPLC methods compared favorably for naturally contaminated wheat, although the FPIA had a bias towards overestimation. Studies on the presence of DON analogs (acetylated derivatives and glucuronides) in cereal grains suggest these ‘masked’ mycotoxins may be present [15]. This may have been a factor, however further studies with the same antibody/tracer combination suggested that the matrix itself can cause overestimation [16]. It seems logical that both factors: cross-reactivity to related toxin analogs and unrelated matrix effects may be able to confound FPIA, as they can with other immunoassays.

Lippolis *et al*. optimized a FPIA for DON in durum wheat kernels, semolina, and pasta [16]. To address matrix effects the amount of toxin found in contaminated samples was determined after subtracting the amount calculated to be due to the matrix effect. The amount due to the matrix was calculated from a total of 150 measurements encompassing 10 different cultivars of durum wheat. Similar experiments indicated that the matrix effects of semolina and pasta were lower than that of durum wheat. The limit of quantitation was 0.10 μg DON/g for the three matrices. Samples were spiked over the range from 0.25 to 1.75 μg/g. Recoveries averaged 98, 102, and 101% from the wheat, semolina, and pasta, respectively. The FPIA was compared to a reference HPLC method for detecting DON in a total of 83 samples, with a good correlation (r > 0.995). The authors suggest the method is suitable for rapid quantitative determination of DON in these products at existing regulatory levels.

### 2.5. Zearalenone

Zearalenone (ZEN) and several of it’s congeners are estrogenic compounds that are often produced by some of the same species of fungi that produce DON. FPIA were initially described for detection of ZEN and related compounds in maize [17], using a ZEN-fluorescein tracer (Table 1). The method had a limit of detection of 0.1 μg ZEN/g maize. However, as with the DON assay described above, the response of the assay was related to the length of the tracer incubation. The method compared well to an HPLC reference method, with a correlation coefficient of *r*^2^ = 0.976. The same group followed up with an examination of an additional five monoclonal antibodies using a handheld instrument [18]. A total of 6 ZEN congeners were tested for each antibody. These included ZEN, zearalanone (ZAN), α-zearalenone (α-ZEOL), β-zearalenol (β-ZEOL), α-zearalanone (α-ZAOL), and β-zearalanone (β-ZAOL). As was the case with DON, marked differences were observed between the antibodies in the kinetics of the competition reaction, most notably in the time for assay equilibration. An optimum assay was obtained using an antibody that rapidly reached equilibrium (2 min), with an IC_50_ for ZEN of 135 ng/mL of the added standard. Because the volume of standard added to the test mixture (0.02 mL) was low compared to the total volume (1.04 mL), the actual concentration of ZEN in the test solution at the IC_50_ corresponded to 2.6 ng/mL. The assay showed cross reactivity to: α-ZAOL (130%), β-ZAOL (101%), ZAN (75%), α-ZEOL (67%), and β-ZEOL (46%), relative to ZEN (100%). The effect of ZEN-fluorescein tracers having different linkages (2, 3, and 6-carbons) between the toxin and the fluorophore were examined by Chun *et al*. [19]. In that report the tracer with the longest bridge was chosen for use in a FPIA for corn. Assays, excluding the extraction step, took less than 2 min per sample. The detection limit was 137 μg/kg, with a detection range of 150 to 1000 μg/kg. Recovery from corn spiked over the range from 50 to 100 μg/kg averaged 106.4 ± 12.5%. When compared to a reference HPLC method the coefficient of determination (*r*^2^) between FPIA and the HPLC method was 0.72 for 70 naturally contaminated cereal products.

## 3. Matrix Effects and Data Treatment

The mycotoxin FPIAs described above have dealt with the presence of matrix effects in various ways. Generally matrix effects in immunoassays can be controlled either through dilution, cleanup, matrix matched calibration curves, or data normalization. The simplest of these, dilution of the sample extract, is usually the method of choice provided the FPIA is of sufficient sensitivity that the additional dilution does not adversely affect the operating range of the assay. Cleanup and/or concentration of sample extracts are usually not as desirable, because this eliminates two of the primary advantages of FPIA: speed and simplicity. It also incurs additional costs. However, for difficult matrices or assays requiring high sensitivity it remains an option. Matrix matching can be accomplished by preparing the calibration standards in extract from a ‘toxin-free’ sample [19]. The underlying assumption with matrix matching is that the matrix of the toxin-free sample is a good mimic for the matrix that will be tested. The same assumption can be used in various forms of data normalization.

Normalization can take several forms. For ELISAs data is typically normalized to allow the comparison of the relative color development (absorbance) between assays conducted on different microtiter plates, or on different days. In this case the minimum color development for ELISAs is ‘0’ (no color), so the normalization is simple: take the observed absorbance and divide by the absorbance maximum and multiply by 100%. Another form of normalization, discussed above for DON in wheat products is the subtraction of the amount of ‘toxin’ found in a control (toxin-free) sample from that found in the unknown sample [16]. This assumes that the matrix effect is constant and not associated with the amount of toxin present. A third form of normalization is to treat the data so as to render their representation linear, for example using a logit-log treatment [19].

**Figure 4:**
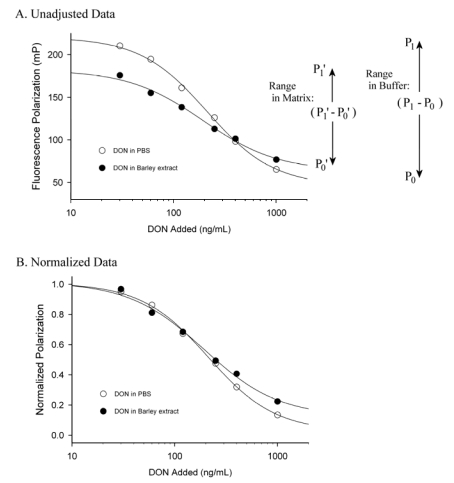
Representation of matrix effects for a DON FPIA.

For FPIA the minimum signal (having no antibody present) is not zero, but rather the signal of the tracer in the absence of antibody. For many fluorescein-based tracers this typically is around 35 to 50 mP. For this reason the normalization should accommodate changes in the lower, as well as the upper, ends of the signal range. For FPIA the lower end of the signal range is that of the tracer alone (P_0_) and the maximum end of the signal range is that of the tracer in the presence of the antibody and absence of the toxin (P_1_). This is best described schematically, as shown in Figure 4. Data falling between the maximum (P_1_) and minimum (P_0_) values can be normalized by the equation *Y*_obs_ = (P_obs_ - P_0_)/(P_1_ - P_0_), where *Y*_obs_ is the normalized polarization of the test sample and P_obs_ is the observed polarization of the test sample (7). This treatment is useful, because matrix effects in FPIA often cause a decrease in the signal range, characterized by both a rise in the minimum polarization and a fall in the maximum polarization (Figure 4a). The signal from sample in a matrix can be normalized to accommodate for the decrease in signal range caused by the presence of the matrix as follows. First, the data for standards in buffer can be collected and normalized using the equation above, so that they are scaled in the range between 0 and 1. Next the analyst can collect a minimum polarization value (P_0_’) by measuring the response of the tracer alone (in matrix lacking toxin), and collect a maximum polarization value (P_1_’) by measuring the response of tracer in the presence of antibody in the same matrix. Next, samples containing unknown levels of toxin can be tested and normalized by the equation Y_obs_’= (P_obs_’ - P_0_’)/(P_1_’ - P_0_’), where Y_obs_’ is the normalized polarization of the test sample and P_obs_’ is the observed polarization of the test sample. By scaling both the standards (in buffer) and samples (in matrix) to the same range (0 to 1) the calibration curve for the standards in buffer can be used to calculate the toxin content of the samples. The effect of this data treatment is seen in Figure 4b. It is apparent that scaling the data can reduce the magnitude of the matrix effect, but does not altogether eliminate it, as evidenced by the disparity between the two curves at higher DON levels in Figure 4b. Care must be used, however, because this treatment is based upon the assumption that the matrix effect from the unknown samples is similar to that of the blank matrix. As such it should be used only when the analyst can normalize against a blank matrix of similar composition to that expected from the samples.

## 4. Conclusions

The number of applications of FPIA to mycotoxin analysis continues to grow. The benefits of FPIA include the elimination of steps to separate bound and free labels, giving the potential for very rapid immunoassays. The reported FPIA are indeed rapid, with most taking from 2 to 15 min to complete. Important assay parameters such as speed and sensitivity are dependent upon not only the quality of the antibody and tracer used, but also upon how they interact in competition with the toxin. Selection of the appropriate antibody and tracer combination can yield rapid and sensitive mycotoxin assays. Although sometimes touted as being free from matrix effects, it is apparent that FPIA, as with other immunoassays, can be affected by the sample matrix. Apart from using the most sensitive antibody and tracer combination available, matrix effects can be controlled using a number of established procedures ranging from simple dilution to matrix-matched calibration. Many of the aforementioned mycotoxin FPIA have been tested and compared favorably to HPLC reference methods, supporting the contention that they may be useful quantitative screening assays. Several existing FPIA instruments are field portable. However, the solution-based nature of the FPIA suggests that, at least in this regard, they may be less easy to use in field settings than lateral flow devices, many of which do not require sequential addition of reagents. Although the FPIA are very simple assays, the logical progression for the technology will be automation of the assay steps to further improve ease of use and reduce operator-associated variablity.
